# The Green Revolution shaped the population structure of the rice pathogen *Xanthomonas oryzae* pv. *oryzae*

**DOI:** 10.1038/s41396-019-0545-2

**Published:** 2019-10-30

**Authors:** Ian Lorenzo Quibod, Genelou Atieza-Grande, Eula Gems Oreiro, Denice Palmos, Marian Hanna Nguyen, Sapphire Thea Coronejo, Ei Ei Aung, Cipto Nugroho, Veronica Roman-Reyna, Maria Ruby Burgos, Pauline Capistrano, Sylvestre G. Dossa, Geoffrey Onaga, Cynthia Saloma, Casiana Vera Cruz, Ricardo Oliva

**Affiliations:** 1 Rice Breeding Platform, International Rice Research Institute, DAPO Box 7777, Metro Manila, Philippines; 2 Institute of Weed Science, Entomology and Plant Pathology, College of Agriculture and Food Science, University of the Philippines, Los Baños, Philippines; 3 Philippine Genome Center, National Science Complex, University of the Philippines, Diliman, 1101 Quezon City, Philippines; 4 Assessment Institute for Agricultural Technology Southeast Sulawesi, Indonesian Agency for Agricultural Research and Development, Jl. M. Yamin No. 89 Puwatu, Kendari, 93114 Indonesia; 5 Food and Agriculture Organization of the United Nations, Immeuble Bel Espace-Batterie IV, Libreville, Gabon

**Keywords:** Population genetics, Bacterial genomics

## Abstract

The impact of modern agriculture on the evolutionary trajectory of plant pathogens is a central question for crop sustainability. The Green Revolution replaced traditional rice landraces with high-yielding varieties, creating a uniform selection pressure that allows measuring the effect of such intervention. In this study, we analyzed a unique historical pathogen record to assess the impact of a major resistance gene, *Xa4*, in the population structure of *Xanthomonas oryzae* pv. *oryzae* (*Xoo*) collected in the Philippines in a span of 40 years. After the deployment of *Xa4* in the early 1960s, the emergence of virulent pathogen groups was associated with the increasing adoption of rice varieties carrying *Xa4*, which reached 80% of the total planted area. Whole genomes analysis of a representative sample suggested six major pathogen groups with distinctive signatures of selection in genes related to secretion system, cell-wall degradation, lipopolysaccharide production, and detoxification of host defense components. Association genetics also suggested that each population might evolve different mechanisms to adapt to *Xa4*. Interestingly, we found evidence of strong selective sweep affecting several populations in the mid-1980s, suggesting a major bottleneck that coincides with the peak of *Xa4* deployment in the archipelago. Our study highlights how modern agricultural practices facilitate the adaptation of pathogens to overcome the effects of standard crop improvement efforts.

## Introduction

The emergence of plant pathogens in agricultural ecosystems represents a threat to food security. Selection for underrepresented virulent clones has become a hallmark of modern agriculture [1]. While natural selective processes affecting the biology of plant pathogens have been defined, the contribution of human interventions in shaping pathogen populations in agricultural systems is poorly understood. The exponential growth of monocultures during the Green Revolution, as a response to increasing human population, has given some opportunities to understand the effect of directional selection on biotrophic pathogens with a high reproduction rate.

In the Philippines as many other countries in Asia, rice is a staple food. Early archeological records of rice production date back to sometime after 1500 B.C. [2], but the first signs of crop selection did not start until 1901 [3]. For the most part of the 20th century, farmers used locally adapted landraces that were grown in relative isolation from other parts of Asia. The situation changed in the early 1960s after the International Rice Research Institute (IRRI) was established and the country emerged as a natural laboratory for testing rice varieties during the Green Revolution. This is where semi-dwarf high-yielding varieties, such as IR8 were first bred, tested, and rapidly spread across Asia, replacing traditional landraces [4]. Nevertheless, IR8 was soon found susceptible to bacterial blight caused by *Xanthomonas oryzae* pv. *oryzae* (*Xoo*). In 1967, breeders released IR20 carrying *Xa4*, a bacterial blight resistance gene located at chromosome 11 [5]. The genomic region carrying this gene was then frequently used in breeding programs for decades to come. By the mid-80s, *Xa4* has spread in millions of hectares across Asia within IR64, one of the first rice mega varieties developed [4]. *Xa4* appears to encode a cell wall-associated kinase that promotes cellulose synthesis and reinforcement during pathogen attack [6].

The fact that virulent *Xoo* populations emerged in the Philippines after the deployment of *Xa4* opened the case to understand the effect of human interventions in the population structure of a plant pathogen. While other resistance genes (i.e. *xa5*, *xa13*, *Xa7*, or *Xa21*) were used in the breeding programs, their deployment appears to be marginal or not significant compared with *Xa4*. In the current study, we took advantage of a unique historical record from the Philippines archipelago to examine the effect of a major selection process in the population of *Xoo* that occurred during the Green Revolution. We traced the presence of *Xa4* in the overall rice germplasm and showed that continuous deployment reached at least ~80% of the total rice-farming area. This massive selection pressure, in fact, favored the emergence of *Xa4*-overcoming groups. Evidence of selective sweep suggests that several populations were affected during short periods of time. Overall, we documented how modern breeding schemes were instrumental to shape contemporary pathogen populations. This finding is particularly important because it expands our understanding on how to contain emerging pathogens in modern agriculture.

## Material and methods

### Mining of *Xa4* alleles in the 3000 Rice Genomes Project

We used the *Xa4* gene sequences described in Hu et al. [6] and BLASTn [7] in the indica rice genome 93-11 [8]. The physical location of *Xa4* in the 93-11 genome was at locus position 610,197 to 615,135 base pair (bp) (Os9311_04g012750). We used this information to extract the gene sequences of *Xa4* in the 3000 rice panel stored in the Rice single nucleotide polymorphisms (SNP)-seek Database [9]. The genes were translated into amino acid sequences and aligned using MUSCLE [10]. We further focused on the 45th position of *Xa4* and assessed allele variants (Additional Data S1). The alleles observed at that position: D/D, E/E, D/E, and stop codon. The glycine residue (D/D) is predicted to be associated with the resistance phenotype [6].

### Evaluation of *Xa4* activity in a 1.2K diversity panel

To characterize the rice diversity panel, a total of 1263 accessions were grown in the quarantine area at the IRRI plot field. All plants were inoculated with three strains of *Xoo* from different races. The isolates used were PXO61 (Race 1), PXO86 (Race 2), and PXO99A (Race 6). The experiment consisted of three replications in a randomized design. IRBB near-isogenic lines (Table S1) were used as controls. Due to the varying maturity of the accessions, staggered planting was performed. Bacteria were grown for 72 h at 28 °C on modified Wakimoto’s medium [0.5 g of Ca(NO_3_)_2_·4H_2_, 0.82 g of Na_2_HPO_4_, 5 g of peptone, 20 g of sucrose, 300 g of potato 15 g agar per liter of water]. These were suspended in distilled water, adjusted to 10^9^ CFU/ml, and used as inoculums. Inoculation was done at maximum tillering stage (45–50 days after sowing) via leaf-clip method as described by Mew et al. [11]. Disease assessment was scored by lesion length in centimeters 14 days post inoculation (dpi) and highlighted varieties with disease reaction as resistant (R < 5 cm) or susceptible (S > 15 cm).

### Assessment of *Xa* genes in modern rice varieties in the Philippines

To determine the varietal share or proportion of area planted with released varieties in the Philippines, we first obtained the publicly available data from a consolidated source at the National Seed Quality Control Services and the Philippine Rice Research Institute. Surveys were performed from 1985 to 2009 [12]. We categorized released varieties into *Xa4*-containing and noncontaining varieties. In addition, a total of 109 varieties, released between 1970 and 2010, were selected to detect the presence of *Xa* genes. These varieties include irrigated and lowland ecosystems. Genomic DNA from selected accessions was extracted using the standard CTAB method [13] and was quantified and normalized by ND-1000 NanoDrop spectrophotometer (NanoDrop Technologies; http://www.nanodrop.com) to a concentration of 50 ng/μl. Molecular markers for *Xa4*, *xa5*, *and Xa21* specific alleles were used in PCR to discriminate the *Xa*-containing genotypes as described [14, 15].

### Bacterial strain collection and pathogenicity test

We accessed 1822 randomly collected *Xoo* live cultures maintained at IRRI, some of these records were reevaluated from Quibod et al. [16] or are part of recent collections. The metadata includes isolate code, isolate phenotype, collection site, and year of collection (Additional Data S2). All strains were randomly isolated from naturally infected epidemics occurring from 1972 to 2015 in different regions in the Philippines. The race of each strain was determined based on the pathogenicity reactions to differential varieties carrying single-resistance genes [17] or a set of the near-isogenic lines. A complete list of IRBB lines is shown in at Table S1. Strains collected were cultured and revived using modified Wakimoto’s medium.

For this study, we used 91 *Xoo* strains from different races and locations for subsequent pathogenicity tests (Additional Data S3). We phenotypically recharacterized the 91 *Xoo* strains based on seven known resistance genes (Table S1) and IR24. The seven lines used are as follows: IRBB4, IRBB5, IRBB7, IRBB10, IRBB13, IRBB14, and IRBB21. A split plot design with rice cultivar as the main plot and bacterial strain as the subplot was employed in virulence tests. The selected five fully expanded leaves in each of the three plants were inoculated as described above. *Xoo* was grown and maintained following the methods described above. Disease reactions were scored by measuring the expressed lesion length in centimeters at 14 dpi with the following designations: <5 cm = resistant (R), 5–10 cm = moderately resistant (MR), 10–15 cm = moderately susceptible (MS), and >15 cm = susceptible (S).

### Genome sequencing, assembly, annotation, pan-genome, and SNP mining

We selected 80 representative *Xoo* strains from different Philippine *Xoo* races (Additional Data S3) which were isolated across various parts of the Philippines for sequencing. The other *Xoo* strains were already sequenced using PacBio [16, 18, 19]. Ion Proton was used to sequence single-end reads and reads between 1.2 and 2.6 million with mean read length of 158–174 per strain were acquired. The *Xoo* cultures were grown overnight at 30 °C and extraction of DNA was done using the Easy-DNA kit (Invitrogen, USA) following the manufacturers’ protocol. De-novo assembly and error read correction were performed using MIRA 3.4.1 [20]. As suggested by Baez-Ortega et al. [21], the optimized parameter group of importance for the assemblies are as follows: Align (-AL:mo = 19, -AL:ms = 15), Assembly (-AS:nop = 5, -AS:rbl = 3, -AS:urd = no, -AS:ardml = 200, -AS:mrl = 40), Clipping (-CL:qcmq = 20), and MISC (-MI:lcs = 500). The resulting contigs from the assembly were evaluated using the quality assessment tool QUAST 2.3 [22] and aligned to the reference genomes of *Xoo* strains PXO99A and PXO86. In addition, contigs with a total length of less than 500 bp were removed. The number of contigs obtained ranges from 803 to 1188 with a mean total size of 4.7 Mb. The assembled genome coverage was from 36.5× to 81.8×. Furthermore, the final *Xoo* contigs were oriented through ABACAS 1.1 [23] using the nearest complete *Xoo* genome strains as the reference according to Fig. 2a. Prokka [24] was applied for gene calling and annotation. After annotation, pan-genome analyses were constructed for genes and IGRs through Roary 3.11.0 [25] and Piggy [26], respectively. Core genome alignment and variant calling were performed using Parsnp which is part of the Harvest suite 1.1.2 [27]. The core genome of all the 91 *Xoo* strains is ~3.3 Mb with ~12,900 SNPs.

### Phylogenetic, molecular clock, and population genetic structure analyses

Phylogenetic reconstruction was evaluated using the core genome alignment and executed via a maximum-likelihood approach implemented in RaxML 8.2.9 [28]. Statistical confidence for each node was passed to 1000 bootstrap run, employing the general time reversal model of nucleotide substitution with the Gamma model of rate heterogeneity. With the same parameters, we also performed a global phylogenetic analysis using SNPs from strains listed in Additional Data S3.To perform the molecular clock analysis, metadata of each PX-A lineage strains were included. SNPs with putative recombination events were masked. We used TempEst v1.5.1 [29] to estimate the divergence rate via a root-to-tip branch length linear regression. BEAST v1.8.4 [30] was used for a Bayesian time-calibrated phylogenetic reconstruction with five replicates under a strict clock and three different population models. The nucleotide substitution configuration was selected with the general time-reversible model. Each run was allowed to continue for 100 million iterations, sampling the posterior every 1000th iteration. Run logs were combined using LogCombiner v1.8.4. Trees were summarized using TreeAnnotator v1.8.4. The effective sample size for all the parameters of interest was greater than 200. Each branch of maximum clade credibility tree was reported at greater than 50% posterior probability. The trees produced in the analysis were visualized using the R package ggtree [31].

To understand the level of substructuring we performed a hierarchical-clustering approach implemented in HeirBAPS [32] using the SNP data. Estimated BAPS clusters were obtained through five independent runs through two levels of nested clustering with a prior upper bound interval of 10–30. The nested genetic populations of the first and second levels are 6 and 20, respectively. Clustering was also validated using Principal Component Analysis (data not shown). Using the core genome, we calculated the genome-wide nucleotide diversity (pi), Wright’s fixation index (*F*_ST_), and Tajima’s D with the assistance of the R package PopGenome [33]. Each of the measured genetic analyses used sliding windows of 1, 5, and 10 kb pair at different population structure groups which is also subdivided as before or after the mid-1980s.

### Detection of recombination and selection

ClonalFrameML 1.25 [34] was used to detect recombination events in the *Xoo* core genome alignment obtained from the Harvest suite. In addition, the tree obtained from RAxML was loaded as the starting tree. The standard ClonalFrame model was operated to collect an initial result for inferred recombination with 1000 bootstrap. For selection detection, we assembled a codon alignment from SNPs mapped from the PXO99A genome annotation. Signals for selection were inferred applying FUBAR [35] which is implemented in HyPhy 2.2.4 [36]. For FUBAR parameters, 10 MCMC chains were used, each consisting 1,000,000 iterations. The first 50,000 were discarded as burn-in and 300 postburn-in samples were drawn with the Dirichlet prior set to 0.5. Evidence of positive selection for each codon was identified at posterior probability ≥ 0.90. Genes with codons under strong-positive selection were grouped into different Gene Ontology (GO) terms using Blast2GO [37]. GO enrichment analysis was performed with the R package clusterProfiler [38] and the *p*-values were corrected for multiple comparisons under Benjamini–Hochberg procedure. Enriched levels have adjusted *p*-value of less than 0.05 and *q*-value of less than 0.05.

### Identification of *Xa4*-associated interaction

Microbial association study was performed using the R package treeWAS [39]. The genotypic data input used was a combination of SNPs and the Roary and Piggy presence–absence matrix. The phenotypic data for the association study were from the lesion length produced in IRBB4. The phenotypic data are used in three ways: (1) binary which is either susceptible (S) or resistant (R), (2) discrete which is susceptible, MS, MR, or resistant, and (3) continuous which is the measured lesion length data in cm. Table S2 shows how the qualitative phenotypic data are grouped according to lesion length data. treeWAS uses three kinds of association test based on population structure correction and these are simultaneous, subsequent, and terminal. A genomic variant is considered linked to *Xa4* if the *p*-value is <0.01 in nine or eight intersecting sets on phenotype-treeWas association. Bonferroni correction was used.

### *Xoo* diversity from an endemic area in the Philippines

To detect the representation of *Xoo* diversity in a field, we established a trapping system by deploying 30 near-isogenic lines with single and multiple *Xa* resistance genes (Table S1) in a farmer’s field where high disease pressure of bacterial blight was constantly observed. This experiment was conducted in two consecutive rice growing periods during the wet season in 2014 and 2015 in Victoria, Laguna. The established field was divided into microplots. Each near-isogenic line was grown in 3-m^2^ microplot area. The experiment was laid in randomized complete design with three replications per line. Rice seedlings were transplanted at 21 days after sowing with 20 × 20 cm planting distance between hills. A total of 45 hills were transplanted per microplot, each hill consisting of three seedlings. IR24, a susceptible line, served as border between microplots with two rows transplanted at the same time as the tested lines. Natural infection of bacterial blight on rice plants was assessed during the peak of the disease between the late tillering to heading growth stages of the plants. The assessment for disease severity was done by randomly selecting seven hills in each microplot. In each hill, five diseased leaves were randomly recorded for the percentage diseased leaf area following the IRRI Standard Evaluation System for Rice 5th edition. Rice leaves were collected in all near-isogenic lines for isolation and strain characterization through pathotyping as mentioned above.

## Results and discussion

### A major resistance gene defined the Green Revolution in the Philippines

Allele frequency of resistance genes tends to experience drastic changes in agriculture compared with natural systems [40]. To estimate the frequency of *Xa4* before the introduction of improved varieties, we investigated the allelic diversity and phenotypic pattern of *Xa4* in two different rice diversity panels representing similar genetic groups. Recently, Hu et al. [6] speculated that residue D^45^ might be associated with the resistance phenotype. We first mined 3000 rice genomes [41] and found that alleles containing such residues are present in more than 62.5% of the accession worldwide (Fig. S1A, B; Additional Data S1). However, when we characterized the phenotypic response of 1263 nonredundant rice landraces to *Xoo* tester strains, less than 1.27% showed phenotypic patterns that are consistent with *Xa4* activity (Fig. S2). This inconsistency suggests that D^45^ might not be associated with resistance in rice, or that an unknown suppressing mechanism in the pathogen prevent the allele from activating immunity. Nevertheless, it is highly likely that landraces used in the Philippines through traditional agriculture maintained resistance alleles of *Xa4* at very low frequency.

Apparently, this situation changed with the rapid introduction and adoption of improved varieties during the Green Revolution. Between 1960 and 2010, more than 100 rice varieties were released in the Philippines’ national system [42]. Using *Xa4*-linked markers, we found that more than 70% of those lines carry a genomic introgression with *Xa4*. In contrast, the proportion of *xa5-* [43] or *Xa21-*containing [44] varieties was not represented during the same period (Fig. S3). While previous reports suggested that *Xa4* strengthens cell-wall integrity and confers beneficial agronomic traits [6, 45], it is unclear whether this effect contributed to its selection during field trials or it was a completely unintentional event [4]. Likewise, farmers’ adoption rates of these varieties followed rapid changes in the landscape. By 1985, nearly 82% of the total rice-cultivated areas in the Philippines were under *Xa4-*containing varieties. Selection pressure of *Xa4* reached a plateau in the late 1980s or early 1990s when the area reached a maximum of 91.5% in 1988 (Fig. S4). While the frequency of these varieties decreased gradually during the next decades, *Xa4* appeared to be prevalent in the landscape (Fig. S4). Temporal and spatial variation of resistance alleles has been described in broad ranges of situations [40]. However, in here we presented a case in which human intervention drove the expansion of improved varieties, causing significant alterations on the allele frequency of a single resistance gene within a semi-isolated environment.

### *Xa4* deployment is associated with a major population shift of *Xoo*

**Fig. 1:**
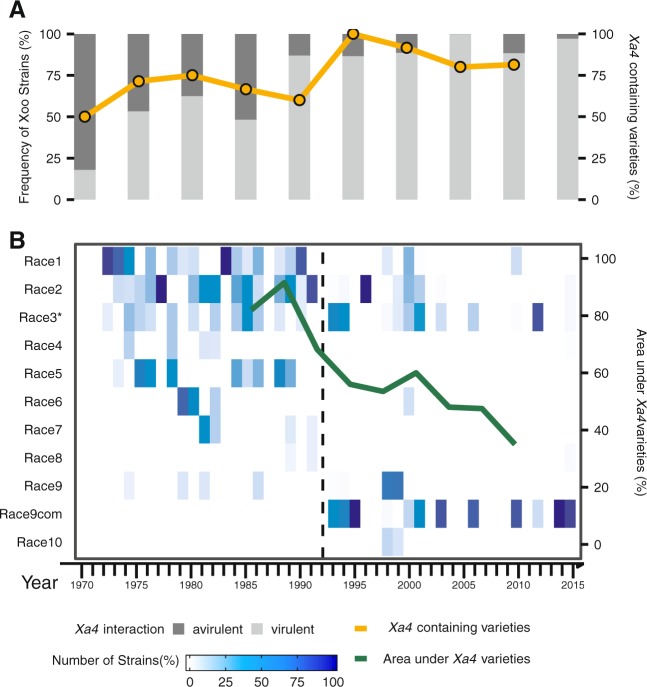
Historical frequency of *Xanthomonas oryzae* pv. *oryzae* (*Xoo*) outbreaks in the Philippines in response to *Xa4* deployment during 45 years. **a** A bar chart centered on the proportion of *Xoo* strains that shows virulent (light gray) or avirulent (dark gray) phenotypes on *Xa4*. The orange solid line constitutes the percentage of released rice varieties containing *Xa4* in the Philippines. **b** A heat map based on the distribution of races collected from 1970 to 2015. The green solid line embedded in the heat map indicates the percentage of area planted with *Xa4*-containing rice varieties from 1985 to 2009 [12]. Furthermore, the dashed black line describes the emergence of Race 9com as the most prevalent race after 1992. Race 9com represents different subgroups: 9a, 9b, 9c, and 9d [66]. The asterisk in Race 3 signifies genetically different populations with the same phenotypic reaction [72]. Phenotypic information from 1822 *Xoo* strains was used (Additional Data S2)

Artificial deployment of a single-dominant gene might generate enough selection to drive significant changes in the population structure of a plant pathogen in short periods of time [46]. To investigate the effect of *Xa4* deployment on *Xoo* populations, we profiled the pathogenicity reaction of 1822 isolates representing disease outbreaks that occurred in the Philippines between 1970 and 2015. Each entry was inoculated in a set of tester rice lines carrying different resistance genes (Table S1). The isolates were characterized into 11 races and four derived groups based on its phenotypic reaction (Additional Data S2). Overall, we found that *Xa4*-overcoming strains increased in frequency and this expansion peaked sometime in the early 90s (Fig. 1a). In the early 1970s, around 20% of field strains were able to grow on *Xa4*. Scientists started noticing a significant increase in the frequency of *Xa4* virulent strains recovered after the release of IR20 [11]. However, it was not until early 1990, which is also the peak of *Xa4* deployment in the archipelago, when the frequency of *Xa4* virulent outbreaks increased dramatically to 84% (Fig. 1a). To further characterize the population shift, we dissected the composition of *Xoo* races recovered before and after the outbreaks in the 90s. Interestingly, we noticed the emergence of a new race complex called 9com (Fig. 1b). Other races with different phenotypic background, such as Race 2 and Race 3, were systematically recovered during the intensification of *Xa4* cultivation across the country (Fig. 1b; Fig. S4). Therefore, it is highly likely that the continuous cultivation of *Xa4*-containing varieties drove the expansion of *Xa4*-overcoming groups in a short period of time. Theoretical models that measure the effect of applying strong selection on field microbes have been described [47]. These models can be used to describe an incidence in which human interventions change the evolutionary trajectory of a plant pathogen [48–50]. For instance, *Yr17*-overcoming strains of yellow rust were only detected in wheat varieties in Northern European countries where those varieties were planted in large areas [48]. A similar scenario can explain the emergence of stem rust Ug99 overcoming *Sr31* in wheat [51] or the spread of Fusarium wilt TR4 in banana [52]. However, our study adds an extra layer of detail by using a traceable record of disease outbreaks in a semi-isolated environment over long periods of time to establish a clear correlation with agricultural deployment.

### *Xoo* populations in the Philippines maintain distinct genomic features

**Fig. 2:**
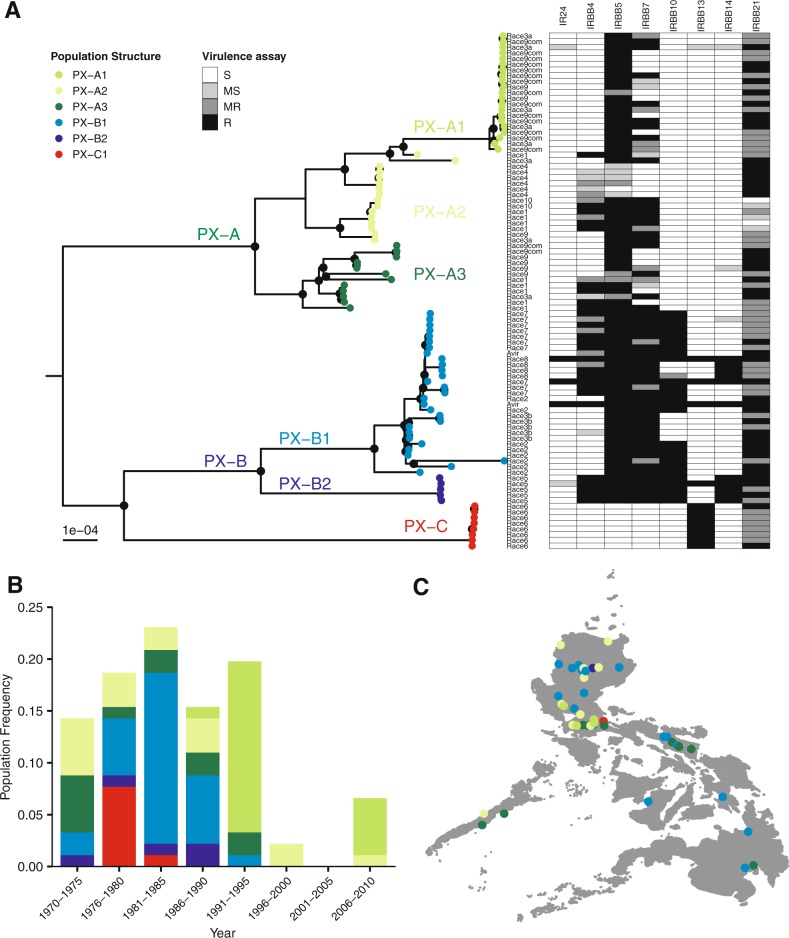
Phylogenetic relationship and population structure of *Xanthomonas oryzae* pv. *oryzae* (*Xoo*) strains collected in the Philippines between 1970 and 2015. **a** A maximum-likelihood phylogenetic tree constructed with core genome alignment using bootstrap of 1000. Black nodes depict bootstrap score of ≥90. Lineage designations PX-A, PX-B, and PX-C were adapted from Quibod et al. [16]. Population PX-A1, PX-A2, PX-A3, PX-B1, PX-B2, and PX-C1 were predicted from BAPS cluster analysis [32]. Race designation is displayed for each strain. The right-side panel shows differential virulence phenotypes based on the reaction to seven near-isogenic lines carrying single *Xa* genes (Table S1). Lesion lengths were averaged across 15 replicates. The reaction is as follows: R resistant, MR moderately resistant, MS moderately susceptible, and S susceptible. **b** Temporal distribution of PX populations isolated in the Philippines between 1970 and 2015. The numbers of strains in each time periods are: 1970–1975 = 13, 1976–1980 = 17, 1981–1985 = 21, 1986–1990 = 14, 1991–1995 = 18, 1996–2000 = 2, 2001–2005 = 0, and 2006–2010 = 6. **c** Spatial distribution of PX populations recovered from different islands in the Philippines archipelago

To further characterize the genetic composition of key *Xoo* groups in the Philippines, we sequenced 91 strains to assess genetic ancestry, population structure, and genomic distribution of coding and noncoding elements (Additional Data S3). The assembled genomes represent ten races isolated across the archipelago during a period of 37 years (Fig. 2a–c). Using a maximum-likelihood analysis, we identified three groups matching reported Philippine *Xanthomonas* lineages, namely PX-A, PX-B, and PX-C [16]. As reported earlier, PX lineages appear to be diverse (Fig. S5) and match ancestral lineages in South Asia [53] and Southeast Asia [54, 55]. Recently, Carpenter et al. [56] used whole-genome analysis to link PX groups with Indian *Xoo* lineages and found a similar pattern.

Population structure analysis based on allele frequencies grouped existing lineages into six modern populations: PX-A1, PX-A2, PX-A3, PX-B1, PX-B2, and PX-C1 (Fig. 2a) that indicate that events of local host adaptation and genetic exchange might follow the putative colonization of the archipelago and elsewhere. As suggested within other bacterial systems [57], we hypothesized that dispensable coding and noncoding regions will be shared by *Xoo* individuals with similar evolutionary histories. To test this hypothesis, we classified the 401,285 predicted open reading frames and 221,849 intergenic regions (IGR) into 15,716 orthologous genes and 11,741 IGR clusters. We plotted all single-copy genes and IGRs that were present in at least two strains and showed that all members of the same population tend to have similar sets of dispensable genomic features as well (Fig. S6A, B).

Pan-genomic dispensable elements across all populations account for 62% of the genes and 55% of the IGRs. However, the number of dispensable elements in each population ranges from 24 to 52% of the genes and 31 to 56% of the IGRs (Figs. S6A, B and  S7), which indicates a highly diverse population. Even though several genes might be missing due to limitations during sequencing and assembly, the trend could indicate similar capabilities in each population as a result of ancestry but also of parallel adaptation to the local host. Furthermore, almost 21% of the pan-genome is comprised of mobile genetic element (MGE) gene clusters. This finding is also aligned with the distribution of transposable elements in *Xoo* [16] as those might play critical roles in spreading fitness-related genes in *Xanthomonas* genomes [58]. Whether or not dispensable genomic features, such as genes or mobile elements, provided alternative functions with selective advantage is unclear [57], but under an evolutionary perspective our data is consistent with the idea that members of the same population share similar genes, ancestry, and might have similar adaptation potential.

### *Xoo* populations show distinct signatures of mutation and recombination

**Fig. 3:**
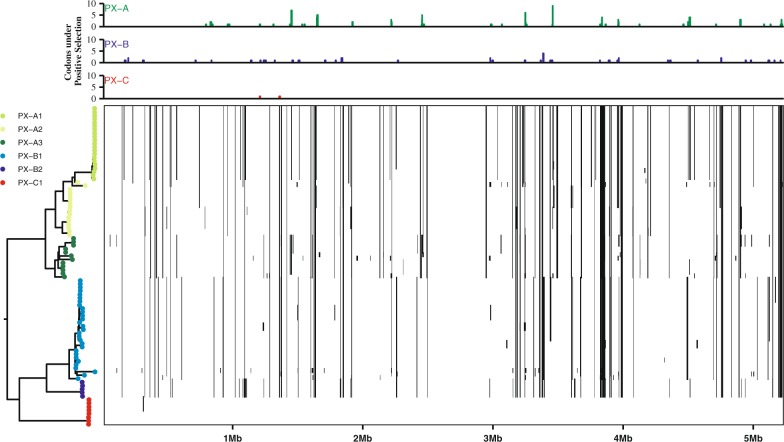
Positive selection and recombination patterns of *Xanthomonas oryzae* pv. *oryzae* (*Xoo*) genomes collected in the Philippines. The left panel represents a maximum-likelihood reconstruction using core genome alignment. The phylogenetic tree was extracted from Fig. 2a. Population PX-A1, PX-A2, PX-A3, PX-B1, PX-B2, and PX-C1 were predicted from BAPS cluster analysis [32]. The bottom right panel shows the predicted recombination events (black lines) in the genome of each strain using a 10 kb window. The top right panel depicts the frequency of codons under positive selection per lineage. Positive selection and recombination events are mapped to the PXO99A genome

**Fig. 4:**
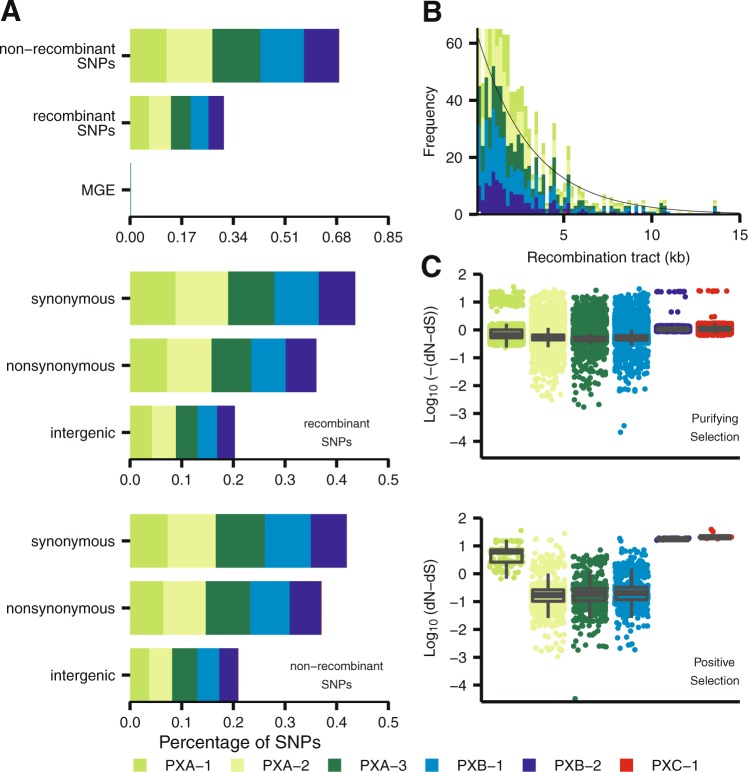
Distribution of single nucleotide polymorphisms (SNP), recombination fragments, and codons under selection in the genome of *Xanthomonas oryzae* pv. *oryzae* (*Xoo*) populations in the Philippines. **a** Percentage of SNPs acquired through mutation, recombination, and mobile genetic elements (MGE) in each of the six major *Xoo* populations. Recombinant and nonrecombinant SNPs are further subdivided into synonymous, nonsynonymous, and intergenic. **b** Distribution showing the length of the recombination event tracts (bp) in the six *Xoo* populations. The black line is a fitted exponential decay curve with a rate of decay of 2.92 × 10^−4^ bp^−1^. **c** Distribution showing the strength of natural selection in each of the six *Xoo* populations using 10,182 codon sites. Selection affecting each codon is classified as either purifying (dN–dS < 0) or positive (dN–dS > 0) selection using FUBAR analysis [35]. To compare the distribution of the dN–dS values affecting every codon in each of the six populations, Wilcoxon rank sum tests were performed. All combinations of the comparison have *p* < 0.0001. Populations PX-A1, PX-A2, PX-A3, PX-B1, PX-B2, and PX-C1 are color-coded

Mutation and recombination are intrinsic forces that influence the evolutionary potential of a pathogen population [1]. To investigate the evolutionary trajectory of *Xoo*, we estimated the rate of mutation and recombination across different PX populations. Overall, the ratio of recombination to mutation (R/θ) showed distinct patterns, suggesting that each PX population has a unique signature, and therefore a unique evolutionary trajectory (Fig. 3; Table S3). R/θ in the sample was 0.452 (95% CI: 0.451–0.453), the average fragment length was 938 bp (95% CI: 937–940 bp), and the divergence of each fragment was 0.006647 (95% CI: 0.006643–0.006651). Thus, genetic variation through mutation was twice more likely to occur than recombination (Fig. 4a), but because each recombination event introduces approximately six substitutions, recombination introduced substitution over mutation is 2.82 (95% CI: 2.81–2.83) times higher within recombinant fragments. These values were similar to those found in Indian *Xoo* populations [53] or what has been reported for other *Xanthomonas* species [59, 60].

Interestingly, *R*/*θ* values show different patterns across PX populations (Figs. 3 and 4a; Table S3). We found that recombination is more frequent in PX-A2, PX-A1, and PX-A3 where the genomes of these groups account for 64% of the overall recombinant SNPs found in genic and IGR (Figs. 3 and 4a, b), compared with PX-B1 and PX-B2 which both account for 35% of recombination signal. A total of 691 recombination events were observed, majority of which involved small recombination fragments with a length of less than 1000 bp. While the highest fragment detected was 13,696 bp, the average recombination fragment length in each population was quite small (Figs. 3 and 4b), suggesting that contribution might be rather ancestral and most likely impact general fitness. For instance, we found a recombinant hotspot in the genomic position between the PXO_02079 and PXO_02062 genes (Fig. S8) which are enriched in components from the restriction-modification system components that are involved in bacterial defense systems, epigenetic-related mechanisms, and recombination and genome rearrangements [61].

On the other hand, genetic variations due to mutation show significant differences across *Xoo* populations (Fig. 3a) with values ranging between 22.95% (PX-A3) and 0.13% (PX-C1). To assess the impact of these mutations in the fitness of each population, we calculated the rate of synonymous and nonsynonymous codon changes (dN–dS ratio) per population (Figs. 3c and  S9A, D) using a rapid hierarchical Bayesian method [35]. While the overall average dN–dS ratio per codon was 1.83 (Fig. S9A), we found signatures of positive selection (*p*-value < 0.05) enriched within genes involved in membrane trafficking, carbohydrate metabolism, proteolysis, and pathogenicity (Fig. S9B, C). Furthermore, we discovered that PX-A populations have significantly higher dN–dS ratios on average than PX-B populations (Fig. S9D). However, genes undergoing selection processes are enriched in populations PX-A2, PX-A3, and PX-B2 (Fig. 4c) which also coincide with the frequency of mutational SNPs (Fig. 4a). Our data support different evolutionary signals across *Xoo* populations in the archipelago. However, whether those changes were shaped by host adaptation or other environmental factors remains unclear.

### *Xoo* appear to evolve different mechanisms to overcome *Xa4*

Plant pathogens evolved multiple strategies to evade or suppress host immunity [62]. To investigate the genetic bases of *Xoo* adaptation to *Xa4*, we performed a phylogenetic-based genome-wide association [39] between SNPs, dispensable genes, and IGRs (Fig. S10) and three phenotypic datasets. Overall, we discovered candidate genetic regions linked to *Xa4* virulence (Fig. S11A). We found 1544 genomic variants (SNP, presence/absence genes, and IGRs) showing significant association with the phenotype (Fig. S10). We focused on SNP-based data because it accounts for 85.16% of the significant hits (*p* ≥ 0.01). Furthermore, a total of 31 *Xa4*-associated SNPs was selected based on 8–9 intersecting sets of phenotype-treeWas association (Fig. S10). These mutations are located within coding regions or in the vicinity of 14 highly targeted genes (Fig. S11A; Additional Data S4). The mutations are likely to cause nonsynonymous substitution, frameshift, or expression polymorphism in known pathogenicity functions such as secretion system, cell-wall degradation, detoxification of reactive oxygen species, and lipopolysaccharide production (Fig. S11C). Interestingly, a number of selected SNP variations were also within genes subjected to purifying selection (Fig. S11B), which might indicate that variations target genes with important virulence functions. Our findings align with reports on other pathogens like *Pseudomonas syringae* which found that the majority of effector families undergo purifying selection as their main evolutionary pathway [63]. Meanwhile, two genes (PXO_01955 and PXO_01954), which are also near the secretion system regulon hrpX and hrpG, showed positively selected SNPs (Fig. S11C). The hrpX and hrpG are crucial genes in the signaling and coordination of virulence effectors inside the host [64]. While the genome-wide association’s resolution is relatively limited due to sample size, it is strong enough to point out to a range of pathogenicity-related regions in the *Xoo* genome. The multiple genetic signals also suggest that *Xoo* might evolve more than one way to adapt to *Xa4*, a strategy that has been suggested for bacterial adaptation to antibiotics [65].

*Xa4* is a wall-associated kinase with pleiotropic effects including cell-wall reinforcement. Incompatible interaction with *Xoo* produces a rapid induction of the gene, leading to a resistance phenotype [6]. *Xoo* likely evades recognition by modulating the components of the secretion system or preventing enzymatic degradation of the host cell. Another alternative is that unknown effector genes actively suppress rice innate immunity. While the identity of *AvrXa4* is still unknown, the forthcoming functional characterization of these candidate genes will bring insights into the specific mechanisms of adaptation to *Xa4*.

### PX-A1 emerge recently and became dominant in the Philippines archipelago

The overall data suggest that PX-A1 emerged during the early 1990s as a response to the accumulative activity of *Xa4* in the Philippines. To estimate the time of the most recent common ancestor of this population, we used a Bayesian model over 10,251 SNPs. Interestingly, the time-scaled tree estimated a quite recent appearance of this population, likely after the 1950s (Fig. S12). Most of the PX-A1 members were detected for the first time during the wet season of 1991–1992 (Fig. 1a, b), suggesting that PX-A1 was already present in the country before the Green Revolution.

During the next two decades, however, PX-A1 became the dominant population in the Philippines (Fig. 1a, b). PX-A1 involves a number of races with similar virulent patterns [66], but it is not clear that all the races are prevailing. If PX-A1 emerges after a recent event of recombination, we speculated that in the absence of a major selection pressure other than *Xa4*, PX-A1 members will maintain the same fitness under local conditions. To assess this question, we used a diversity trapping system consisting of 30 near-isogenic lines planted in a disease endemic area during 2 consecutive years. More than 95% of the strains recovered from symptoms belonged to PX-A1 but were assigned to different races based on their phenotypic reaction with other *Xa* genes (Fig. S13, Table S1). All the recovered races have the capability to overcome *Xa4* but also segregate in response to *Xa7* (Fig. 2a), suggesting that PX-A1 maintains several races with similar fitness in the field.

We looked closely into the region comprising *AvrXa7*, the effector recognized by *Xa7* [67]. We found that the effector lies close to an unstable 20-kb pair region with a high recombination rate (Figs. S8 and S14A). Sequence alignment of this region suggests several events of gene transfer with members from PX-B populations, including *AvrXa7* and the genetic cluster of *XopAA* (Fig. S14A and B). This observation aligned with other reports which link the evolution of *Xoo* effector genes to active recombination events [68]. Probably the strong host selection imposed by *Xa4* drove the expansion of PX-A1 in nature and the absence of other forces (e.g. lack of *Xa7*) reduced the impact of gene conversion on overall fitness to avoid clone selection.

### Genome-wide analysis suggests recent selective sweep in *Xoo*

**Fig. 5:**
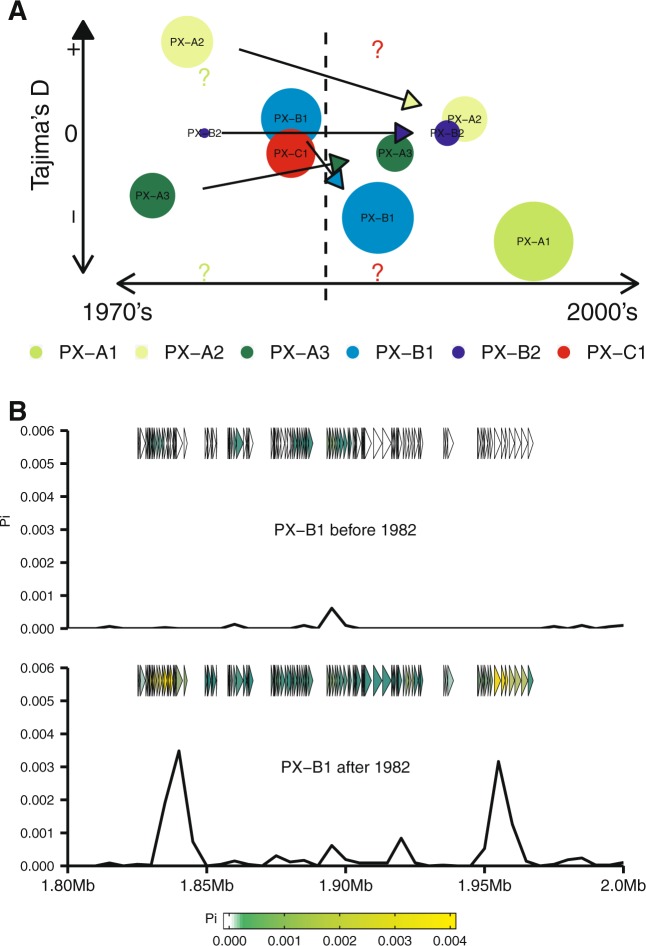
Genome-wide variation in Tajima’s *D* values and nucleotide diversity (pi) in *Xanthomonas oryzae* pv. *oryzae* (*Xoo*) populations in the Philippines during the Green Revolution. **a** Tajima’s D computations of six populations subdivided into epidemics occurring before and after the mid-1980s. The size of the circles represents the number of representative isolates from each population (Table S4). Solid arrow lines illustrate the direction of known *Xoo* demographics. Question marks indicate no representative samples from a particular time point. Timeline is represented in the *X*-axis. Populations PX-A1, PX-A2, PX-A3, PX-B1, PX-B2, and PX-C1 are color-coded. **b** Dramatic change in nucleotide diversity (pi) within a 140 kb fragment from the PX-B1 population collected before and after the mid-1980s. The *Y*-axis represents pi values while the *X*-axis represents the location of each gene (triangle) within the 140 kb fragment. The average values of pi for each gene range from lower diversity (white) to higher diversity (yellow)

Strong directional selection is capable to favor changes in the phenotype of a population within a few generations [69]. This bottleneck hypothesis implies that genetic diversity within each population changed drastically in a short period of time, leaving signatures behind it. We used quantitative data from outbreaks collected before and after the early 1990s to show a significant change in the virulence reaction of strains to *Xa4* compared with *xa5*, *Xa7*, and *Xa10* (Figs. 1 and S3). To test for signatures of demography and selection, we used a genome-wide allele frequency distribution on each population. We found *Xoo* populations showing genome-wide Tajima’s D, Fixation index (*F*_ST_), and nucleotide diversity (pi) values significantly different from the null expectation (Table S4). For instance, pi values in PX-A2, PX-A3, and PX-B1 were significantly higher than the rest of the populations with an average of 19-fold (Table S4). Furthermore, Tajima’s *D* values also support a range of nonrandom events driving the population structure of *Xoo* in the archipelago. We found that PX-A1 (Tajima’s D = −1.9) and PX-B1 (Tajima’s D = −1.8) having significantly lower values of diversity compared with what is expected under neutrality (Table S4), suggesting that *Xoo* populations might experience different evolutionary trajectories, some driven by ancestry but also by stochastic events such as selection, demographic fluctuation, or genetic drift (Fig. 5a). Evidence of these mechanism shaping pathogenicity factors within the genome of barley and wheat pathogens have been reported [70, 71].

**Fig. 6:**
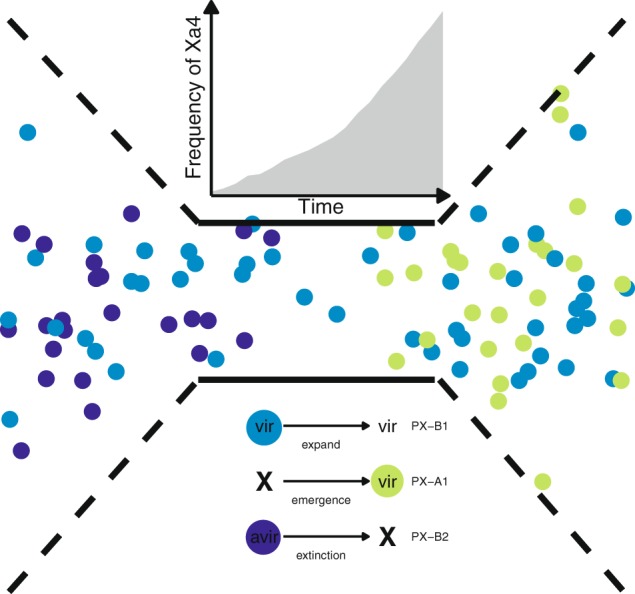
Schematic view representing the effect of a major bottleneck on *Xanthomonas oryzae* pv. *orzyzae* (*Xoo*) populations during the Green Revolution. Before the Green Revolution, *Xa4* activity appears to be present in low frequency within traditional rice landraces. Therefore, a mixture of *Xa4* virulent (vir) and *Xa4* avirulent (avir) populations was expected to occur (colored dots). With the increasing adoption of *Xa4*-containing varieties, a major bottleneck forced avirulent populations to go extinct (dark blue dots) while virulent populations adapted and expanded (light blue dots). Furthermore, previously undetected virulent populations also emerged in the archipelago (green dots)

To investigate if the deployment of *Xa4* caused a selective sweep during the Green Revolution, we used allele frequency distribution in populations before and after the early 1980s and found evidence of strong directional selection (Fig. 5a; Table S5). Apart from PX-A1 negative values, PX-A2 and PX-B1 also appear to experience a substantial reduction of Tajima’s *D* values after the 1980s. However, the removal of genetic diversity in each population might have different contexts. In the case of PX-A1 which emerged after the 1990s, the bottleneck might be recent and directional as it removed genetic variation homogeneously across the genome of this group (Fig. S15). This finding is consistent with our field observations that indicate PX-A1 as the prevalent group in the archipelago. The case of PX-B1 population, on the other hand, matches the emergence of Race 2 soon after *Xa4* was deployed [11]. While selection favored few genotypes that became dominant, the populations quickly expanded across the geography, accumulating neutral mutations. Interestingly, our data showed that the genetic diversity of PX-B1 after the early 1980s was significantly higher and is distributed in particular regions of the genome (Figs. 5b and S15). In both cases, PX-A1 or PX-B1, the data showed nonrandom changes in allele frequency, which is consistent with a dramatic event of selective sweep. This effect is illustrated in Fig. 6. While *Xa4* deployment appears to have a causative effect, we cannot exclude alternative explanations, such as cryptic sampling bias or unknown demographic patterns, that might render similar results.

Coevolution of host and pathogens across agricultural ecosystems is a major concern for long-term strategies of food security. In such a uniform setup, human interventions are likely to increase the magnitude and frequency of emerging virulent pathogens. In this report, we documented a case in which a modern breeding revolution actually shaped the population of a pathogen in a short period of time. This selection force leaves distinct genetic signatures in the genome and causes irreversible changes in the overall fitness of the pathogen. The report provides important insight into the adaptation of pathogens to modern agriculture and advice on the directions of future strategies that include the spatial and temporal deployment of valuable resistance genes.

## Supplementary information

Supplementary Figures and Tables

Additional Data S1

Additional Data S2

Additional Data S3

Additional Data S4

## Data Availability

The sequences produced in this study are stored under BioProject PRJNA525332. Selection analysis results are under Additional Data S4. Genomic files in genbank format are available at https://figshare.com/articles/The_impact_of_the_Green_Revolution_on_the_population_structure_of_rice_pathogens/7842026.
