# Supplementary material for: The Green Revolution shaped the population structure of the rice pathogen Xanthomonas oryzae pv. oryzae
Source: ISME J. 2019 Oct 30;14(2):492–505. doi: 10.1038/s41396-019-0545-2 (PMC6976662; doi:10.1038/s41396-019-0545-2)
Supplement: Supplementary file 1 — Supplementary Figures and Tables [file 41396_2019_545_MOESM1_ESM.pdf]

The Green Revolution shaped the population structure of the rice pathogen *Xanthomonas oryzae* pv. *oryzae*

Ian Lorenzo Quibod[a], Genelou Atienza-Grande[a,b], Eula Gems Oreiro[a], Denice Palmos[c], Marian Hanna Nguyen[a], Sapphire Thea Coronejo[a], Ei Ei Aung[a], Cipto Nugroho[a,d], Veronica Roman-Reyna[a], Maria Ruby Burgos[a], Pauline Capistrano[a], Sylvestre G. Dossa[a,e], Geoffrey Onaga[a], Cynthia Saloma[c], Casiana Vera Cruz[a], Ricardo Oliva[a]

[a]Rice Breeding Platform, International Rice Research Institute, DAPO Box 7777, Metro Manila, Philippines

[b]Institute of Weed Science, Entomology and Plant Pathology, College of Agriculture and Food Science, University of the Philippines Los Baños, Philippines

[c]Philippine Genome Center, National Science Complex, University of the Philippines, Diliman, 1101 Quezon City, Philippines

[d]Assessment Institute for Agricultural Technology Southeast Sulawesi, Indonesian Agency for Agricultural Research and Development, Jl. M. Yamin No.89 Puwatu 93114, Kendari, Indonesia

[e] Food and Agriculture Organization of the United Nations, Immeuble Bel Espace-Batterie IV, Libreville, Gabon

To whom correspondence may be addressed: E-mail: r.oliva@irri.org

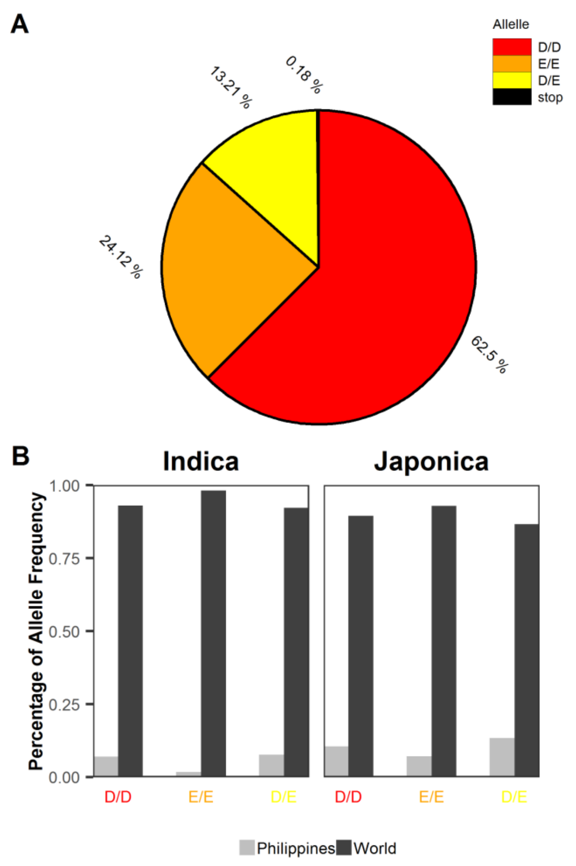

Fig. S1. Distribution of *Xa4* allele frequency at the 45th position. (A) Percentage of different *Xa4* alleles among the 3,000 rice accessions (1). **(B)** Allele frequency of *Xa4* from two major rice subspecies, indica and japonica. Light grey colored bars show rice genotypes collected in the Philippines, while dark grey colored regions represent rice genotypes collected from other sources except Philippines.

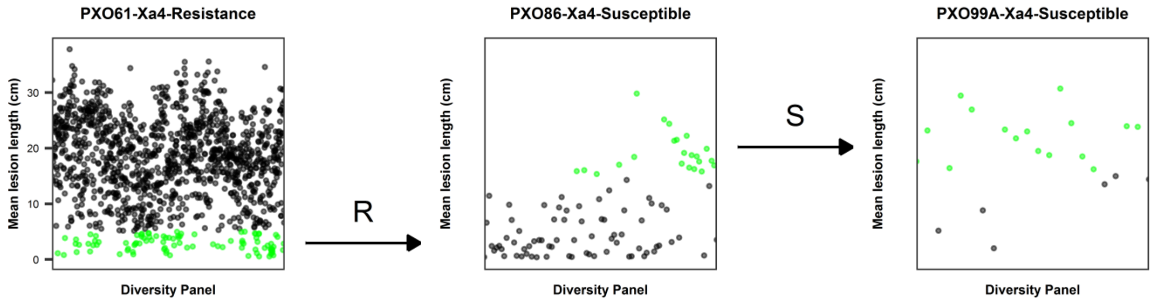

Fig. S2. Potential *Xa4* activity within 1,263 rice landraces after pathogenicity reactions against select *Xanthomonas oryzae* pv. *oryzae* races. All the rice lines were successively challenged with three selected strains to assess the presence of Xa4 activity in the filtered rice panel. The left plot represents the reaction of PXO61 (Race 1), which cannot overcome *Xa4* or *xa5*. To eliminate the presence of most of the genes, only those accessions showing resistance reactions (R) were challenged with the next strain. The middle plot shows the reaction of PXO86 (Race 2), which can overcome *Xa4* but not *xa5*, where only lines with susceptible (S) reactions were advanced further. The right plot shows the reaction of PXO99 (Race 6), which will distinguish the empirical presence of *xa13*. Green dots are the rice panels used for the successive *Xa4* screening, while the black dots were filtered out. Lesion length was measured 14 days post-inoculation and averaged across three replicates. The R and S above the arrow sign denote resistance and susceptibility respectively.

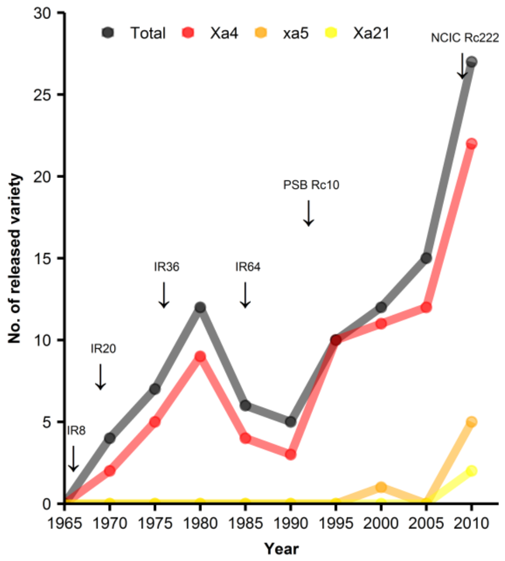

Fig. S3. Number of *Xa4*-containing rice varieties released in the Philippines. The color lines represent the number of varieties containing *Xa4* (red), *xa5* (orange), *Xa21* (yellow), and the overall varieties released (black). The rice varieties printed on top represent popular and widely planted elite varieties. Except for IR8, all of the other mentioned rice lines contain *Xa4*.

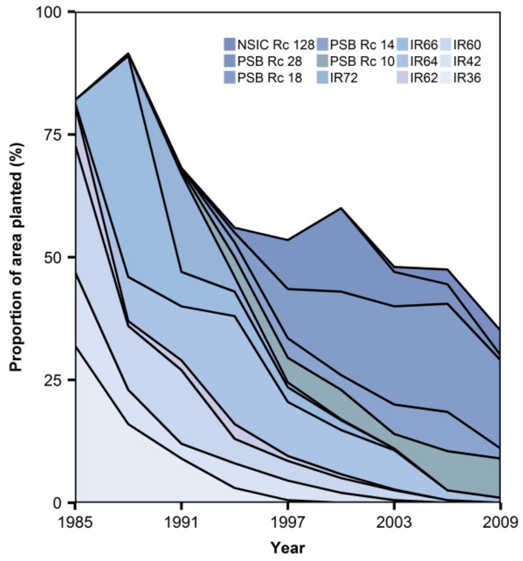

Fig. S4. Proportion of hectares planted with elite rice varieties containing *Xa4* in the Philippines from 1985 to 2009. Data was modified from the paper of Brennan and Malabayabas (2). Blue colors represent varieties containing *Xa4* introgression.

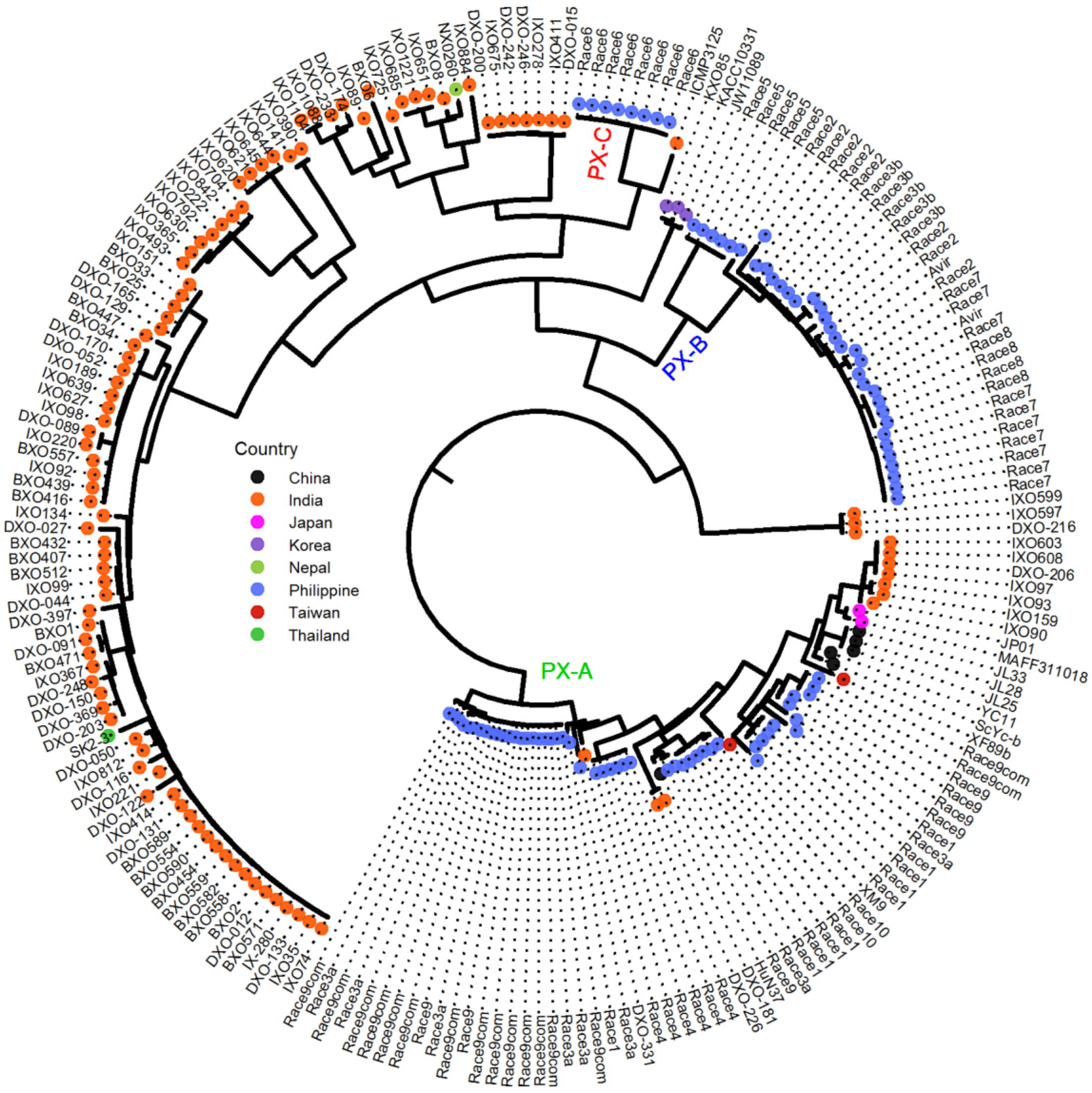

Fig. S5. Phylogentic reconstruction of 208 global *Xanthomonas oryzae* pv. *oryzae* strains. A maximum likelihood tree was constructed with bootstrap of 1000 using 19,706 extracted SNPs. Lineage designations inside the tree are based from Quibod et. al (3). Country of origin and race designation for Philippine strains are displayed. All strains used are listed in Additional Data S3.

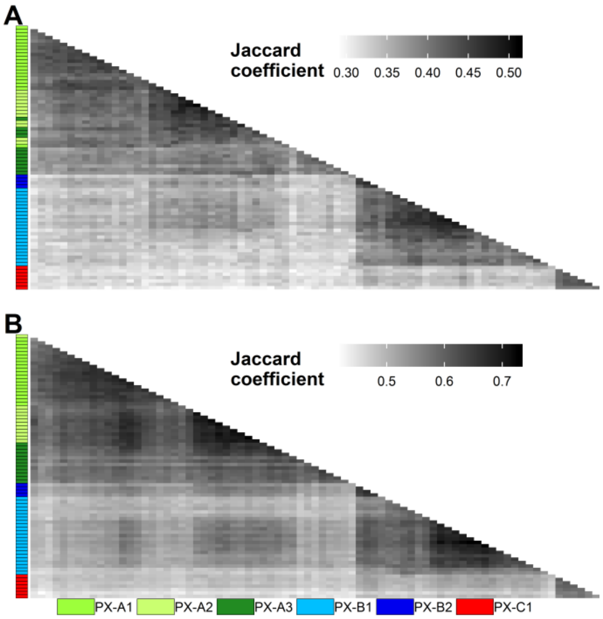

Fig. S6. Pairwise relationship of dispensable pan-genomic content of the *Xanthomonas oryzae* pv. *oryzae* (*Xoo*) reveals high resemblance within populations. (A) A heat map based on 10,043 dispensable single copy genes. **(B)** A heat map based on 6,797dispensable intergenic regions (IGR). A white to black gradient is shown to denote level of similarity across strains based on Jaccard similarity index. Colored block on the right indicates population structure inferred through BAPS (4).

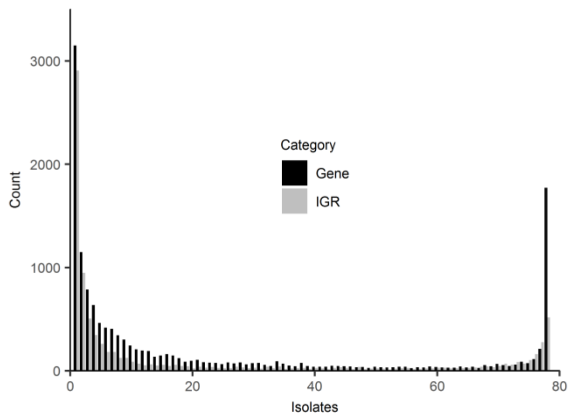

Fig. S7. The pan-genome of *Xanthomonas oryzae* pv. *oryzae* (*Xoo*) are filled with strain-specific contents. Frequency of genes and intergenic regions (IGR) are displayed using 15,716 orthologous gene and 11,741 IGR clusters.

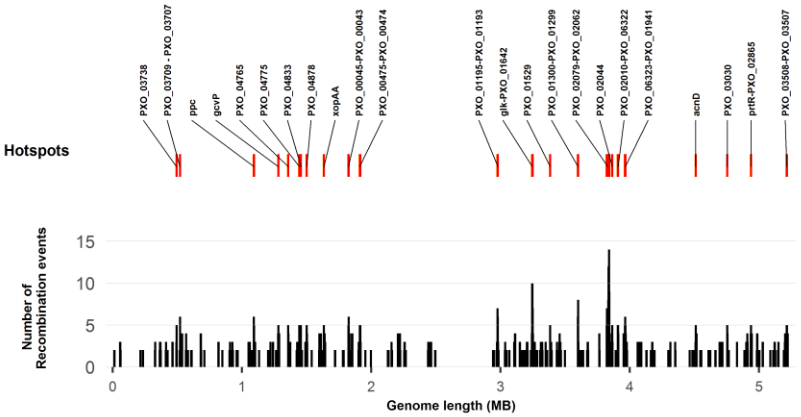

Fig. S8. Recombination hotspots detected in the *Xanthomonas oryzae* pv. *oryzae* (*Xoo*) genome. The plot illustrates the inferred core recombination events mapped in the PXO99A genome. At the top, a total of 26 regions were identified as potential recombination hotspots.

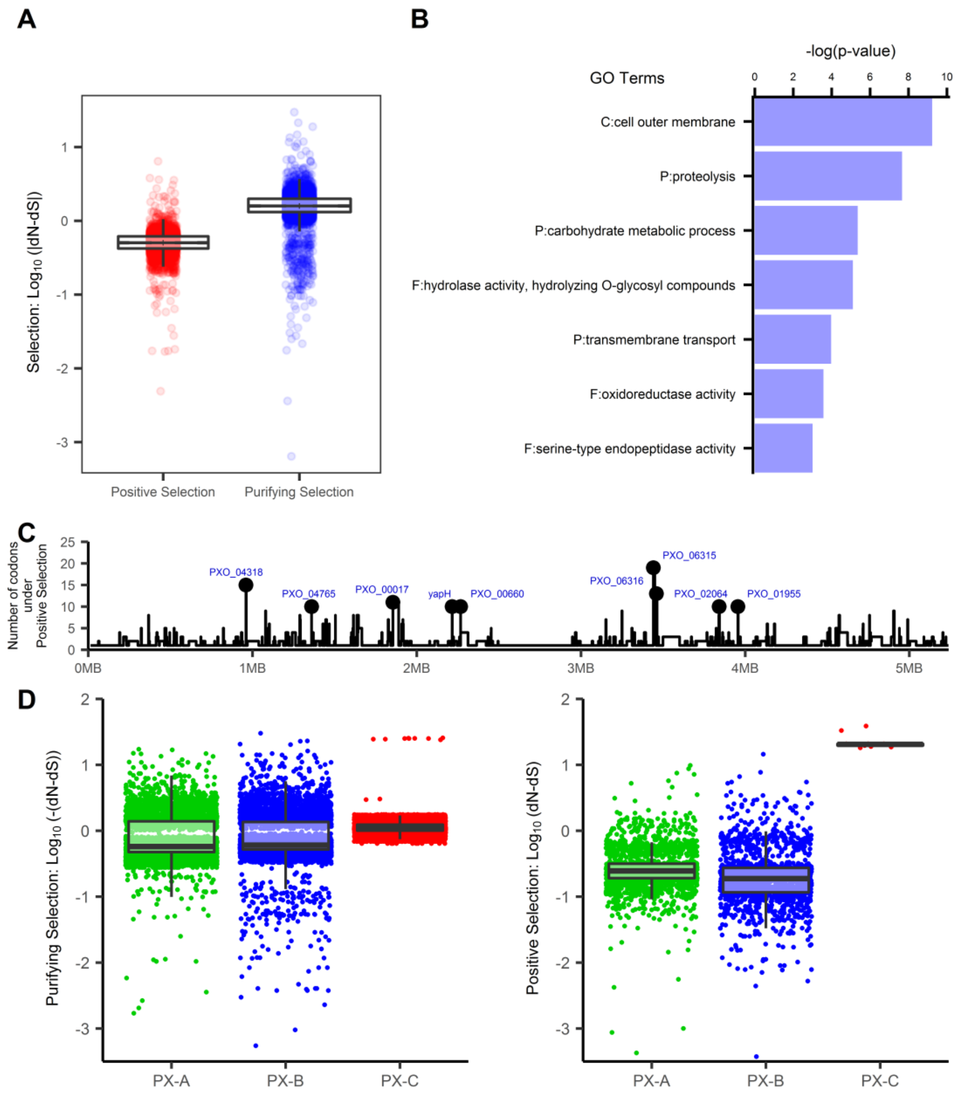

Fig. S9. Signatures of positive selection in the *Xanthomonas oryzae* pv. *oryzae* (*Xoo*) genome using 10,182 codon sites. (A) Distribution showing the strength of natural selection in the codons affected by positive (dN – dS > 0) or purifying (dN – dS <0) selection. The rate of synonymous (dS) and non-synonymous (dN) mutations was calculated using FUBAR analysis (5). **(B)** Gene ontology (GO) enrichment analysis using positively selected genes. Seven GO categories were represented with p-values less than 0.05. **(C)** Distribution of positively selected codons per genes mapped in the PXO99A reference genome. Black point and blue label represent genes with more than ten positively selected codons. **(D)** Distribution showing the strength of natural selection in each of the three lineages using 10,182 codon sites. To compare the distribution of the dN-dS values affecting every codon in each of the three lineages, Wilcoxon rank sum tests were performed. All combination of the comparison has p < 0.0001.

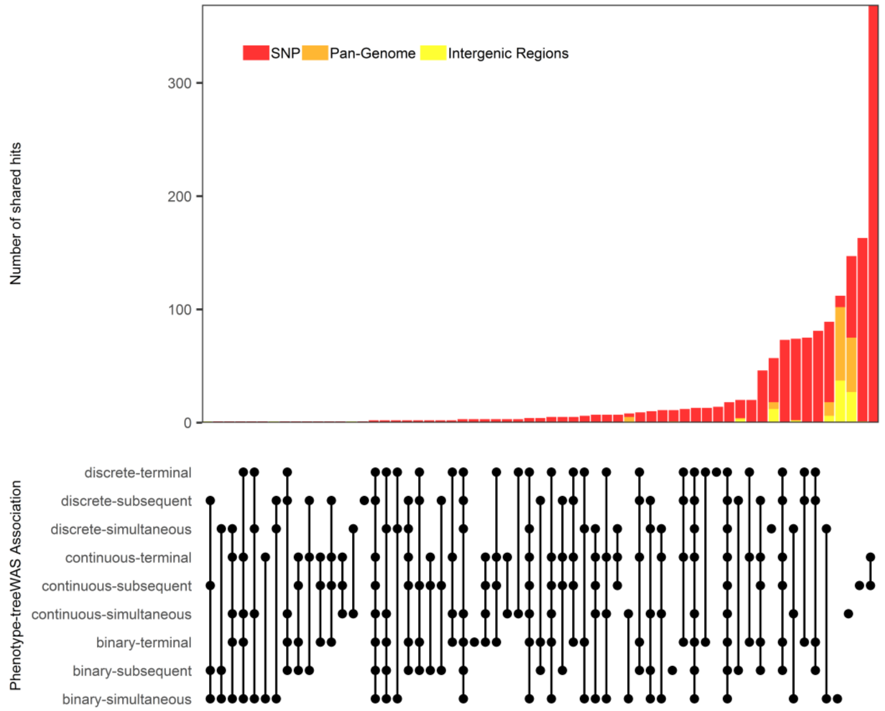

Fig. S10. Intersecting sets of different phenotype data and treeWAS association tests combination were collected to obtain *Xa4* associated genotypes. Three different genotype data were used for the association study, and these are SNPs, and dispensable genes and intergenic regions (IGR). Furthermore, three different types of phenotype data based on lesion length were also used, and these are binary, discrete and continuous (Table S5). Each phenotype and genotype association was tested in terminal, simultaneous, and subsequent test which are implemented in treeWAS (6). Associated genotypes have p-values less than 0.01.

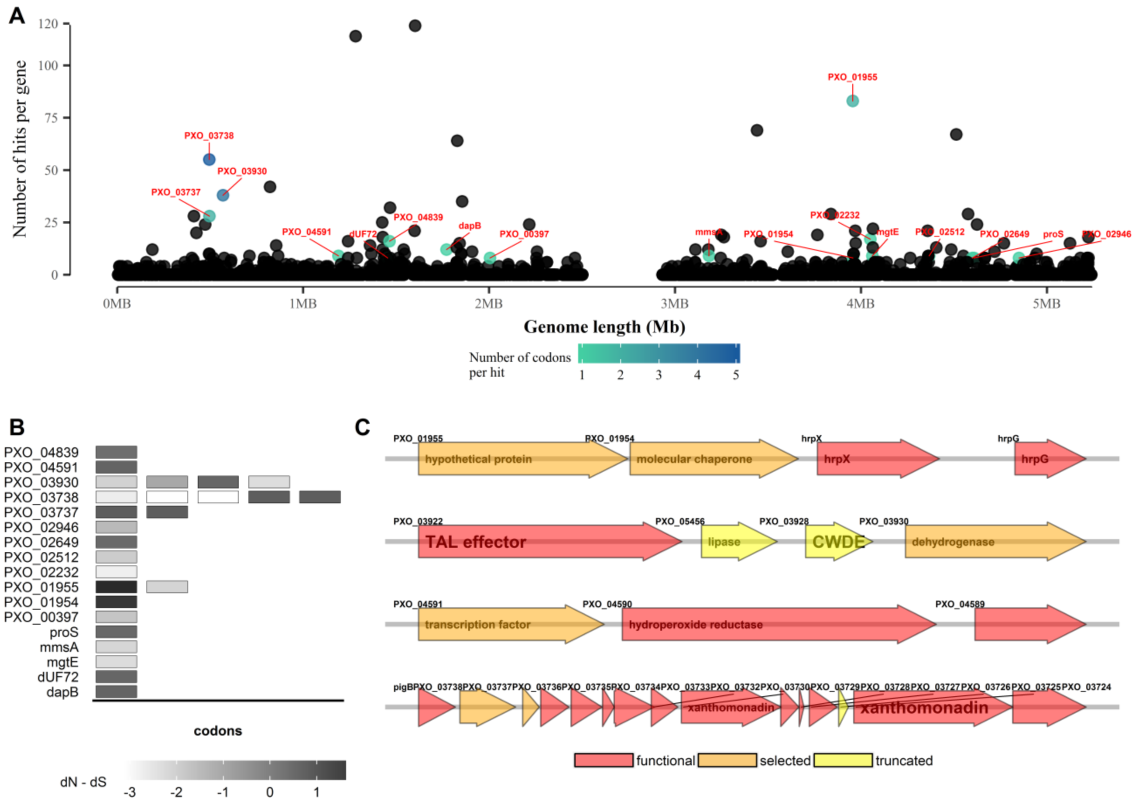

Fig. S11. Multiple genetic factors appear to control the interaction of *Xanthomonas oryzae* pv. *oryzae* (*Xoo*) with *Xa4* resistance gene. (A) Manhattan plot showing the position of SNPs associated with *Xa4* resistance using treeWAS (6). The x-axis serves as the PXO99A genome, while the y-axis indicates the total number of times a SNP within a gene was statistically significant ($p < 0.01$). Hits with varying shades of blue represent the number of SNP position per gene that has strong *Xa4* association. **(B)** Signatures of either positive (dN – dS > 0) or purifying (dN – dS < 0) in codons of genes associated with *Xa4* resistance. The rate of synonymous (dS) and non-synonymous (dN) mutations was calculated using FUBAR analysis (5). **(C)** Example of four independent Xoo gene clusters involved in pathogenicity and associated with *Xa4* resistance. Color code of the genes further identified which are truncated (yellow), functional (red), or carrying SNP with a strong association (orange). The locus tag above each gene is from PXO99A genome.

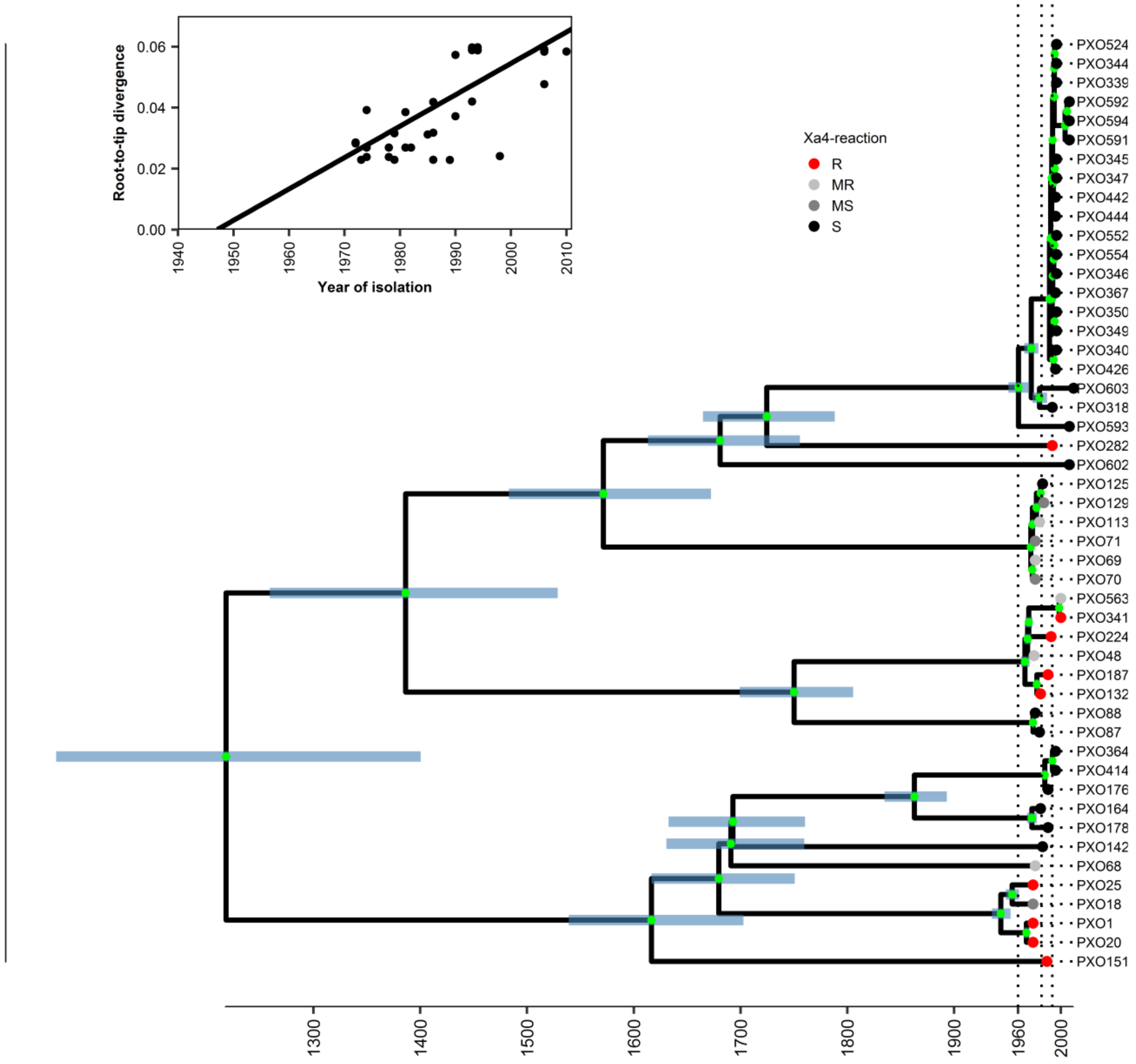

Fig. S12. Temporal phylogenetic relationship *Xanthomonas oryzae* pv. *oryzae* (*Xoo*) of PX-A lineage. Data along the x-axis are in calendar years (CE), and blue bars show 95% highest posterior density (HPD). Posterior probabilities of node positions (P =1) are indicated as green dots. Dashed lines highlight the following years: 1960 (The recent common ancestor of the PX-A1 population), 1982 (adaptation of *Xa4* overcoming strains) and 1992 (emergence of the PX-A1 population). The tip colors are based on different *Xa-4* reactions: R = Resistant, MR = moderately resistant, MS = moderately susceptible, S = susceptible. The inset above the tree shows a linear relationship between the root-to-tip divergence and the year of isolation.

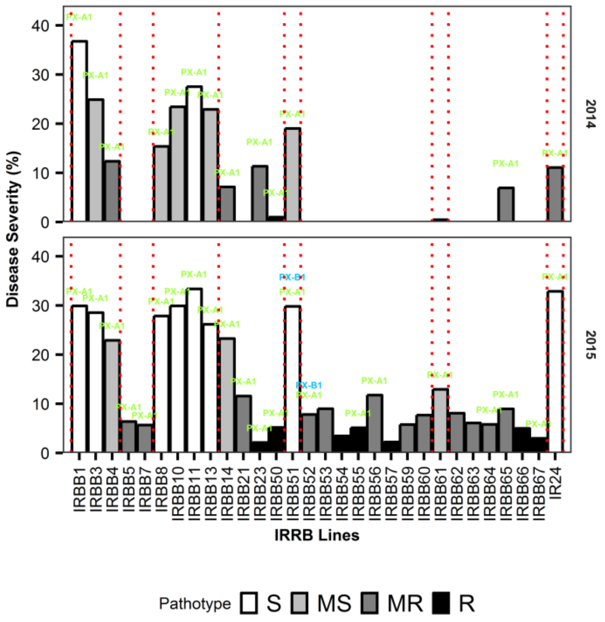

Fig. S13. Field experiment designed to capture *Xanthomonas oryzae* pv. *oryzae* (*Xoo*) from a disease endemic area in the Philippine archipelago. Thirty near-isogenic lines (Table S1) carrying single and multiple combinations of rice *Xa* genes were planted during two consecutive year and disease severity was recorded. *Xoo* strains isolated from infected lesions were pathotyped to identify race and population. The trapping system recovered 95% of members of the PX-A1 (green) dominant population and only 5% of PX-B1 (blue).

A

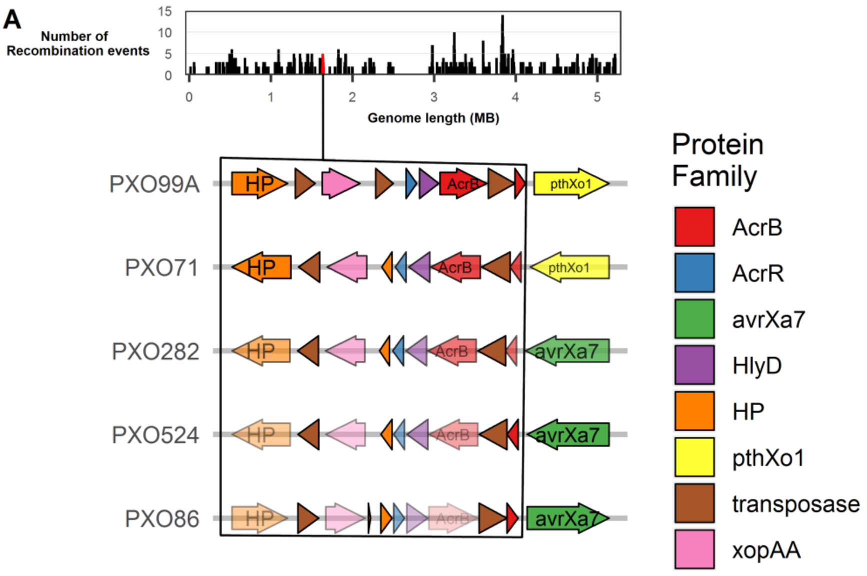

B

Strain	Lineage	Race	AvrXa7
PXO86	B	2	NI-HG-NI-NI-NS-HD-NN-HD-HD-HD-NS-N*-N*!-HD-HD-NS-NS-NN-NN-NI-NG-NN-NI-N*-NS-N*
PXO524	A	9com	NI-HG-NI-NI-NS-HD-NN-HD-HD-HD-NS-N*-N*!-HD-HD-NS-NS-NN-NN-NI-NG-NN-NI-N*-NS-N*
PXO282	A	1	NI-HG-NI-NI-NS-HD-NN-HD-HD-HD-NS-HD-N*!-NI-HD-HD-NN-NS-NN-NN-NG-NN-HD-N*-NS-NS-N*

Fig. S14. A recombination hotspot in *Xanthomonas oryzae* pv. *oryzae* (Xoo) is near TAL effector *Avrxa7* and explain segregation to *Xa7*. (A) Recombination frequency plot in Fig. S7 expanding a region with multiple effectors, including AvrXa7 and XopAA. The transparency of the gene arrows denotes different haplotypes of the gene. **(B)** The table shows the repeat variable di-residue (RVD) sequence of two different alleles of *AvrXa7*.

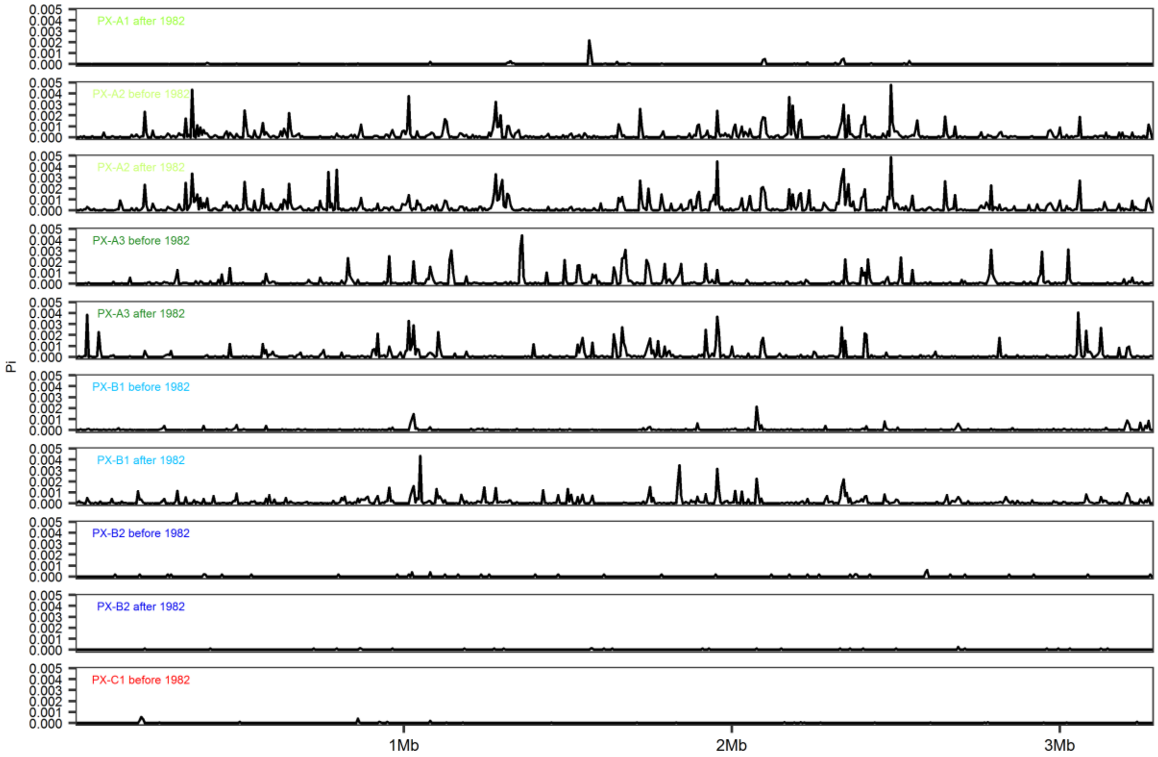

Fig. S15. Genome-wide nucleotide diversity (pi) of the *Xanthomonas oryzae* pv. *oryzae* (*Xoo*) population before and after 1982. A sliding-window of 5-kb regions partitioned the *Xoo* core genome. Colored texts on the top- left indicate population structure inferred through BAPS (4).

Table S1. Near isogenic lines (NILs) with single and pyramided bacterial blight resistance *Xa* genes in IR24 cultivar background deployed in hotspot at Victoria, Laguna (2014-2015).

Line	Designation	R-genes
1	IRBB1	*Xa1*
2	IRBB3	*Xa3*
3	IRBB4	*Xa4*
4	IRBB5	*xa5*
5	IRBB7	*Xa7*
6	IRBB8	*xa8*
7	IRBB10	*Xa10*
8	IRBB11	*Xa11*
9	IRBB13	*xa13*
10	IRBB14	*Xa14*
11	IRBB21	*Xa21*
12	IRBB23	*Xa23*
13	IRBB50	*Xa4+xa5*
14	IRBB51	*Xa4+xa13*
15	IRBB52	*Xa4+Xa21*
16	IRBB53	*xa5+xa13*
17	IRBB54	*xa5+Xa21*
18	IRBB55	*xa13+Xa21*
19	IRBB56	*Xa4+xa5+xa13*
20	IRBB57	*Xa4+xa5+Xa21*
22	IRBB59	*xa5+xa13+Xa21*
23	IRBB60	*Xa4+xa5+xa13+Xa21*
24	IRBB61	*Xa4+xa5+Xa7*
25	IRBB62	*Xa4+Xa7+Xa21*
26	IRBB63	*xa5+Xa7+xa13*
27	IRBB64	*Xa4+xa5+Xa7+Xa21*
28	IRBB65	*Xa4+Xa7+xa13+Xa21*
29	IRBB66	*Xa4+xa5+Xa7+xa13+Xa21*
30	IRBB67	*Xa4+Xa7*
31	IR24	*Xa18*

Table S2. Different lesion length code was used for genome-wide association studies.

	**Qualitative**		**Quantitative**
	binary	discrete	continuous
**Lesion Length**	< 10 cm = R	< 5 cm = R	original lesion length data
		5 - 10 cm = MR	
	> 10 cm = S	10 - 15 cm = MS	
		> 15 cm = S	

Table S3. Contribution of recombination to the diversity of the six *Xanthomonas oryzae* pv. *oryzae* (*Xoo*) populations.

Population	mean length of imports (δ)	average distance of the imports (ν)	Ratio of recombination and mutation rates (R/θ)	relative effect of recombination and mutation (r/m)
PX-A1	4043	0.00276	0.197	2.20
PX-A2	1722	0.00399	0.617	4.24
PX-A3	1542	0.00772	0.320	3.81
PX-B1	1097	0.00724	0.466	3.70
PX-B2	931	0.00738	0.453	3.12
PX-C1	199	0.0547	0.103	1.12

Table S4. Average genome-wide nucleotide diversity (pi), population divergence analysis (F_{ST}), and Tajima's D of the *Xanthomonas oryzae* pv. *oryzae* (*Xoo*) population at different sliding windows.

All Position

	pi (nucleotide diversity)	F_{ST}	Tajima's D
PX-A1	1.39E-05	0.943846	-1.944179
PX-A2	0.000278093	0.85695	0.392306
PX-A3	0.00022758	0.879597	-0.819007
PX-B1	0.000112499	0.922715	-1.878834
PX-B2	9.11E-06	0.95322	-0.9999833
PX-C1	5.02E-06	0.962933	-0.3648163

1k Sliding Window

	pi (nucleotide diversity)	F_{ST}	Tajima's D
PX-A1	1.39415E-05	0.71476	-1.115345
PX-A2	0.000278111	0.663161	0.1781522
PX-A3	0.000227595	0.679982	-0.4268851
PX-B1	0.000112485	0.697978	-0.8756919
PX-B2	9.113E-06	0.716261	-0.6564108
PX-C1	5.023E-06	0.720527	-0.2232263

5k Sliding Window

	pi (nucleotide diversity)	F_{ST}	Tajima's D
PX-A1	1.39E-05	0.922143	-1.16914
PX-A2	0.000278242	0.843133	0.1756824
PX-A3	0.000227733	0.862644	-0.5259881
PX-B1	0.000112553	0.897428	-0.9918145
PX-B2	9.12E-06	0.926382	-0.6603761
PX-C1	5.03E-06	0.935114	-0.4095269

10k Sliding Window

	pi (nucleotide diversity)	F_{ST}	Tajima's D
PX-A1	1.39E-05	0.936882	-1.12595
PX-A2	0.000278242	0.855744	0.1965846
PX-A3	0.000227733	0.872992	-0.6146116
PX-B1	0.000112553	0.911115	-1.081192
PX-B2	9.12E-06	0.942857	-0.6700273
PX-C1	5.03E-06	0.950277	-0.4745619

Table S5. Average genome-wide nucleotide diversity (pi), population divergence analysis (F_{ST}), and Tajima's D of the *Xanthomonas oryzae* pv. *oryzae* (Xoo) population before and after 1982 at different sliding windows.

All Position

	pi (nucleotide diversity)	F_{ST}	Tajima's D
PXA-1 after 1982	1.39E-05	0.9401222	-1.944179
PXA-2 before 1982	0.000228789	0.852494	1.631902
PXA-2 after 1982	0.000324836	0.8147981	0.2465082
PXA-3 before 1982	0.000184447	0.874281	-1.121367
PXA-3 after 1982	1.80E-04	0.8768651	-0.3600715
PXB-1 before 1982	4.51E-05	0.9317693	0.260873
PXB-1 after 1982	0.000157913	0.8918009	-1.519627
PXB-2 before 1982	1.31E-05	0.9446601	0
PXB-2 after1982	6.07E-06	0.9469943	0
PXC-1 before 1982	5.02E-06	0.9627039	-0.3648163

1kb Sliding Window

	pi (nucleotide diversity)	F_{ST}	Tajima's D
PXA-1 after 1982	1.39E-05	7.15E-01	-1.12E+00
PXA-2 before 1982	2.29E-04	6.66E-01	9.56E-01
PXA-2 after 1982	3.25E-04	6.42E-01	1.51E-01
PXA-3 before 1982	1.84E-04	6.80E-01	-5.17E-01
PXA-3 after 1982	1.80E-04	6.83E-01	-3.06E-01
PXB-1 before 1982	4.51E-05	7.04E-01	2.97E-02
PXB-1 after 1982	1.58E-04	6.83E-01	-7.93E-01
PXB-2 before 1982	1.31E-05	7.13E-01	0.00E+00
PXB-2 after1982	6.08E-06	7.14E-01	0.00E+00
PXC-1 before 1982	5.02E-06	7.23E-01	-2.23E-01

5kb Sliding Window

	pi (nucleotide diversity)	F_{ST}	Tajima's D
PXA-1 after 1982	1.39E-05	9.20E-01	-1.16914
PXA-2 before 1982	2.29E-04	8.41E-01	9.87E-01
PXA-2 after 1982	3.25E-04	8.11E-01	1.45E-01
PXA-3 before 1982	1.85E-04	8.60E-01	-5.40E-01
PXA-3 after 1982	1.80E-04	8.68E-01	-3.66E-01
PXB-1 before 1982	4.51E-05	9.03E-01	-8.39E-02
PXB-1 after 1982	1.58E-04	8.71E-01	-8.92E-01
PXB-2 before 1982	1.31E-05	9.17E-01	0.00E+00
PXB-2 after1982	6.08E-06	9.20E-01	0.00E+00
PXC-1 before 1982	5.03E-06	9.36E-01	-4.10E-01

10kb Sliding Window

	pi (nucleotide diversity)	F_{ST}	Tajima's D
PXA-1 after 1982	1.39E-05	9.34E-01	-1.13E+00
PXA-2 before 1982	2.29E-04	8.55E-01	1.04E+00
PXA-2 after 1982	3.25E-04	8.20E-01	1.51E-01
PXA-3 before 1982	1.85E-04	8.71E-01	-5.94E-01
PXA-3 after 1982	1.80E-04	8.78E-01	-4.00E-01
PXB-1 before 1982	4.51E-05	9.17E-01	-1.01E-01
PXB-1 after 1982	1.58E-04	8.83E-01	-9.73E-01
PXB-2 before 1982	1.31E-05	9.32E-01	0.00E+00
PXB-2 after1982	6.08E-06	9.36E-01	0.00E+00
PXC-1 before 1982	5.03E-06	9.50E-01	-4.75E-01

Captions for additional data

Additional Data S1. A list of rice varieties with different *Xa4* alleles

Additional Data S2. Metadata of all *Xanthomonas oryzae* pv. *oryzae* isolates IRRI has collected from 1970 -2015.

Additional Data S3. General information of the all *Xanthomonas oryzae* pv. *oryzae* (*Xoo*) strains used in this study. The first sheet contains Philippine *Xoo* strains representing 11 major races and 4 derived groups. Included information in the list are collection site, year of isolation, total number of contigs, genome size in bp, number of predicted ORF and the coverage. The second sheet contains *Xoo* strains from other countries**.** The list is comprised of country of origin, number of contigs, genome size in bp and source of the data (reference).

Additional Data S4. Distribution of site-specific dN–dS values across different *Xanthomonas oryzae* pv. *oryzae* (*Xoo*) population. The file includes nine sheets of selection data from the overall *Xoo* Philippines, Lineages (PX-A, PX-B, PX-C) and population structure (PX-A1, PX-A2, PX-A3, PX-B1, PX-B2, PX-C1). For complete header description, check the header description sheet.

Reference

1. W. Wang, *et al.*, Genomic variation in 3,010 diverse accessions of Asian cultivated rice. *Nature* **557**, 43–49 (2018).

2. J. P. Brennan, A. Malabayabas, Australian Centre for International Agricultural Research, *International Rice Research Institute's contribution to rice varietal yield improvement in South-East Asia* (Australian Centre for International Agricultural Research, 2011) (February 23, 2019).

3. I. L. Quibod, *et al.*, Effector Diversification Contributes to *Xanthomonas oryzae* pv. *oryzae* Phenotypic Adaptation in a Semi-Isolated Environment. *Sci. Rep.* **6**, 34137 (2016).

4. L. Cheng, T. R. Connor, J. Siren, D. M. Aanensen, J. Corander, Hierarchical and Spatially Explicit Clustering of DNA Sequences with BAPS Software. *Mol. Biol. Evol.* **30**, 1224–1228 (2013).

5. B. Murrell, *et al.*, FUBAR: A Fast, Unconstrained Bayesian AppRoximation for Inferring Selection. *Mol. Biol. Evol.* **30**, 1196–1205 (2013).

6. C. Collins, X. Didelot, A phylogenetic method to perform genome-wide association studies in microbes that accounts for population structure and recombination. *PLOS Comput. Biol.* **14**, e1005958 (2018).
